# *Granulicatella adiacens*, an unusual causative agent in chronic dacryocystitis

**DOI:** 10.1186/s12348-015-0043-2

**Published:** 2015-04-14

**Authors:** Cristy A Ku, Blake Forcina, Paul Rocco LaSala, John Nguyen

**Affiliations:** Robert C. Byrd Health Sciences Center, West Virginia University School of Medicine, Morgantown, WV 26505 USA; Department of Ophthalmology, West Virginia University Eye Institute, One Stadium Drive, Morgantown, WV 26505 USA; Department of Pathology, West Virginia University, Morgantown, WV 26505 USA

**Keywords:** *Granulicatella adiacens*, Chronic, Dacryocystitis, Dacryocystorhinostomy, Nasolacrimal duct obstruction

## Abstract

**Background:**

*Granulicatella adiacens*, a recent taxonomic addition, is a commensal organism of the oral, gastrointestinal, and urogenital tracts and is rarely encountered in the orbit and eye.

**Findings:**

We present a 46-year-old Caucasian woman with chronic dacryocystitis who underwent an external dacryocystorhinostomy and was found to have *G. adiacens*.

**Conclusions:**

This is an unusual causative organism isolated in the nasolacrimal system and, to our knowledge, the first reported case of chronic dacryocystitis associated with *G. adiacens.*

## Findings

### Introduction

Acquired nasolacrimal duct obstruction (NLDO), a common cause of epiphora and dacryocystitis, is typically managed with dacryocystorhinostomy (DCR). Even in cases without signs of infection, positive bacterial cultures are often obtained from the lacrimal sac [1]. Gram-positive bacteria - *Staphylococcus epidermidis*, *Staphylococcus aureus*, and *Streptococcus pneumoniae* - are most frequently identified [1]. We herein describe a rare case of *Granulicatella adiacens*, isolated from a patient with chronic dacryocystitis.

### Case report

A 46-year-old Caucasian woman complained of constant right eye tearing for 6 months, and it had worsened to cause blurry vision and interfered with wearing contact lens. She had intermittent pressure pain in the right medial canthus that improved after treatment with tobramycin-dexamethasone drops. Past history included deviated septum, anxiety, mitral valve prolapse, and venous insufficiency of the legs, and her medications were calcium and multi-vitamins. Visual acuity was 20/20 in each eye. Confrontation visual fields, extraocular motility, and pupillary exam were normal. The intraocular pressure was 15 mmHg in each eye. Pooling of clear tears was noted on a normal-appearing right lower lid without an enlarged, erythematous, or tender lacrimal sac. Nasolacrimal dilation and irrigation revealed complete reflux of clear saline, and the remainder of the ocular exam was unremarkable.

**Figure 1:**
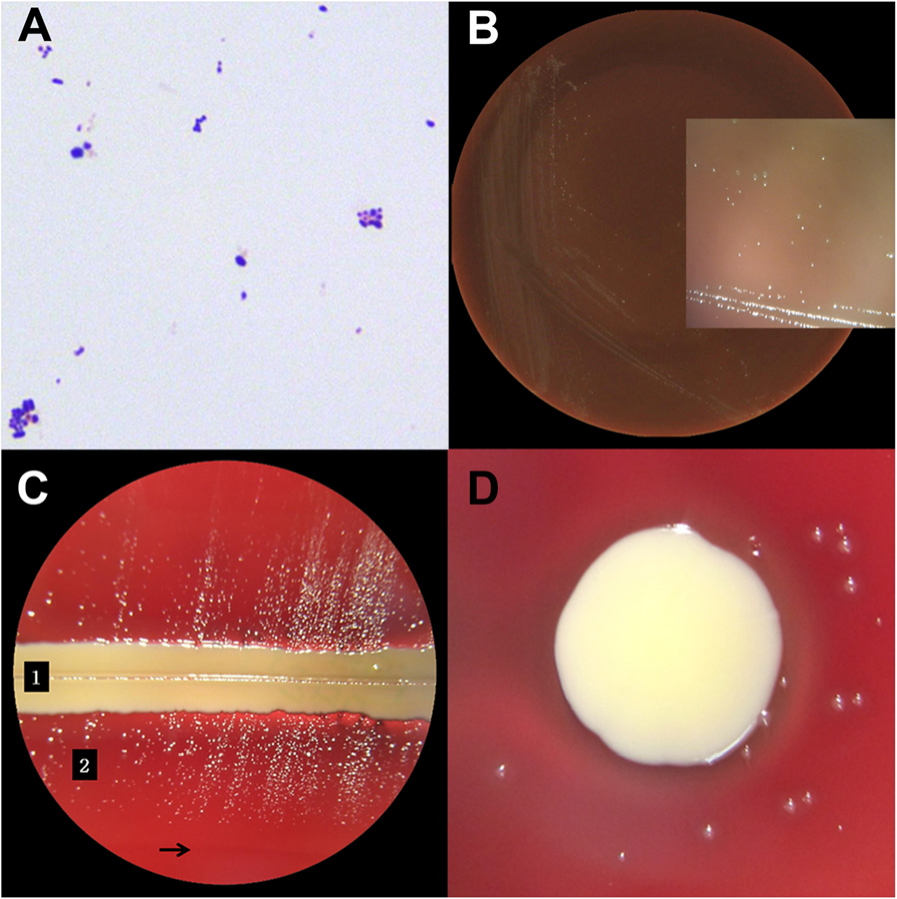
**Results of Gram stain and cultures. (A)** Gram stain showing Gram-positive cocci in pairs and clusters, and pleomorphic coccobacilli (×1,000). **(B)** *Granulicatella adiacens* growing after 72 h at 37°C on a chocolate agar plate as 2- to 3-mm colonies (inset). **(C)** *G. adiacens* growing after 72 h at 37°C on sheep blood agar along a streak of *Staphylococcus aureus* (1) as 1- to 2-mm pinpoint satellite colonies (2), within the zone of beta-hemolysis (arrow). **(D)** 1 to 2-mm pinpoint satellite colonies of *G. adiacens* within the beta-hemolytic zone of a large *Staphylococcus aureus* colony.

The patient underwent an uneventful external dacryocystorhinostomy with placement of Crawford stent. Milky purulent material, found upon entering the lacrimal sac, was sent for Gram stain and culture. The Gram stain showed Gram-positive cocci in pairs and clusters and pleomorphic coccobacilli (Figure 1A). The patient was discharged with oral cephalexin 250 mg four times daily for 5 days along with erythromycin ointment and tobramycin-dexamethasone drops. Routine cultures initially revealed suspected *Granulicatella*/*Abiotrophia* species based on biochemical phenotype, good growth on chocolate agar, and colony satellitosis on sheep blood agar within beta-hemolysis zones of co-inoculated *Staphylococcus aureus* (Figure 1B,C,D). The isolate was subsequently identified as *G. adiacens* with intermediate susceptibility to penicillin (MIC = 0.25 mcg/mL). The patient had an unremarkable postoperative course with resolution of tearing and pain symptoms, and the stent was removed at 3 months follow-up.

### Discussion

Common isolates from dacryocystitis include Gram-positive bacteria (*Staphylococcus aureus* and *Streptococcus pneumoniae*) and Gram-negative bacteria (*Haemophilus influenzae*, *Serratia marcescens*, and *Pseudomonas aeruginosa*) [2]. Anaerobic organisms have also been reported in as much as 16% of isolates, with the most common as *Bacteroides*, as well as *Peptostreptococcus*, *Propionibacterium*, *Prevotella*, and *Fusobacterium*. Furthermore, polymicrobial infection was seen in 45% of cases [2]. Our case was unique due to the presence of a single nutritionally variant streptococcal species.

*Granulicatella adiacens*, *Granulicatella elegans*, *Granulicatella balaenopterae*, and *Abiotrophia defectiva* are nutritionally variant streptococcal (NVS) species, first described and termed in the early 1960s due to their fastidious growth requirements with a streptococcal morphology. Since then, NVS species were found to share less than 10% homology to other streptococcal species [3]. Additionally, although first described as streptococci, these Gram-positive bacteria are pleomorphic with morphologies ranging from cocci, coccobacilli, to rod-shaped cells, depending on the growth medium. The distinct phylogenetics and nutritional requirements of these bacteria relative to *Streptococcus* species led to the creation of the *Abiotrophia* genus, which was later further subdivided into the two genera *Granulicatella* and *Abiotrophia* [3]. *G. adiacens* are alpha- or gamma-hemolytic, catalase-negative, facultative anaerobic, nutritionally complex Gram-positive bacteria and typically require pyridoxal- or l-cysteine-supplemented chocolate or blood agar, or may also grow in satellite colonies around other Gram-positive and Gram-negative bacteria that secrete pyridoxal [3].

*G. adiacens* is part of the normal oral, gastrointestinal, and urogenital tract flora [3]. Clinically, *G. adiacens* is associated with up to 2.3% of cases of streptococcal bacteremia and up to 5% of cases of streptococcal endocarditis. *G. adiacens* is more common than *A. defectiva* and much more common than *G. elegans*, as an etiologic agent of bacterial endocarditis [3]. In the eye and orbit, *G. adiacens* has been sporadically isolated in a case of orbital abscess secondary to trauma [4] and in several cases of infectious crystalline keratopathies and corneal ulcers following penetrating keratoplasty [3,5]. *A. defectiva* has also been implicated in cases of keratitis and ulcers following penetrating keratoplasty and post-cataract surgery endophthalmitis [3,6]. To our knowledge, there has been no report case of chronic dacryocystitis involving *G. adiacens* or other NVS.

The unusual finding of *G. adiacens* in our patient may explain the chronicity of the symptoms. While *G. adiacens* is typically non-virulent, the use of topical corticosteroid-antibiotic combinations may alter the bacterial flora of the lacrimal sac, resulting in susceptibility to infection, as seen in cases of infectious crystalline keratopathies [3,5]. Co-infection with *Staphylococcus aureus* or other streptococcal species has also been speculated to contribute to the growth of *G. adiacens* in vivo [3]; however, we did not find other organisms in our case. Most NVS are moderately susceptible to penicillins, clindamycin, chloramphenicol, erythromycin, rifampin, and vancomycin and variably susceptible to cephalosporins [7]. While these antimicrobial agents provide adequate coverage for common isolates of dacryocystitis, a combination therapy of penicillins and aminoglycoside is recommended for serious *G. adiacens* systemic infection.

## Consent

Written informed consent was obtained from the patient for the publication of this report and any accompanying images.

## Abbreviations

DCR: dacryocystorhinostomy
NLDO: nasolacrimal duct obstruction
NVS: nutritionally variant streptococcal
